# Dental Nomenclature

**Published:** 1867-10

**Authors:** L. C. Ingersoll

**Affiliations:** Keokuk, Iowa


### Original Communications.

### DENTAL NOMENCLATURE.

BY DR. L. C. INGERSOLL, KEOKUK, IOWA.

Read before the Iowa State Dental Society, July, 1867.

IN making this report on DENTAL NOMENCLATURE, I have named some principles of nomenclature, I have made comparison of scientific words in Chemistry and Dentistry with these standards, I have criticised the use of a number of words commonly occurring in our Dental Literature, I have suggested some modifications of terms, and the cautious introduction of new words; and, lastly, I have protested against cumbering and mystifying Dental Science and Literature with strange and unmeaning words.

Words are the repositories and vehicles of thoughts. Words contain ideas and transmit them through the ear, the eye, or the blind man's touch to the mind. By words, the thoughts of the ancients came down to us. Words are the resurrection bodies of their ideas. In words we embalm our own thoughts, our own conceptions of things for posterity. The pen and the press are busy, day and night, everywhere where words are uttered or thoughts are breathed, to render them in imperishable forms for after generations.

Dentistry is the latest born child of Science. To Dental nomenclature is entrusted the naming of the facts, principles, tissues, structures, forces, and functions which are involved in the science. No sooner was this world created, with its grand array of classed and clustered things, than Adam was made a *nomenclator*. Reptiles crawled to him for a name. Insects whispered in his ear for a name. The first animal cry, of whatever kind, was for a name. It is not a matter of little importance in the creation of a new science, arraying before the world a series of newly discovered facts, that they be properly named, that each should be individualized by a name, that every newly discovered principle, and every infant thought conceived by truth should be appropriately named from birth. Principles never die; facts are eternal. For these reasons they should be truthfully named. Nomenclature, therefore, lies at the very foundation of the exact sciences.

I cannot expect in a brief paper on Dental Nomenclature to do more than to preface a broad and deep subject which demands a library volume from the most critical and scholarly minds of our profession. I am sensible, too, that so grand an achievement for our Dental literature cannot be compassed by any assembled body of the Dental profession, however respectable in numbers and talents, less than the representative whole, the great and honored AMERICAN DENTAL SOCIETY.

Here the reform can but be initiated. Its progress must be through the received channels of professional opinion, where the coin of our Dental literature is stamped, and where philological weights and measures shall give their syllabic test.

Science demands exactness of terms. This demand is increased in proportion to the similarity and the variety of the facts and principles developed. Chemistry, which in its progress developed so rapidly an astonishing number of elemental facts, suffered greatly in its infancy for want of a suitable nomenclature. In 1789, the French chemists Lavoisier and Berthollet and their associates, revised the nomenclature of the science, banishing the barbarous and fanciful names which had been introduced, and instead, giving significant and meaning names to all newly discovered substances, leaving only the longest known of simple substances to be recognized by their old names.

It is greatly to be regretted that some elementary substances prevailing everywhere in nature should have been permitted to travel so far on their pilgrimage along the track of time without appropriate, descriptive names. *Magnetism* is an example of this class of words. Perhaps it would be foolish now to change the name of that peculiar property of the lode-stone. It was called magnetism for the simple reason that the ferruginous ore having this property of imparting attractive power and polarity to iron and steel was first found in a locality in Asia Minor, known as Magnesia, called by the Greeks *Magnes*. So also did that tasteless, inodorous powder, sold in the shops as magnesia, derive its name from the fact that the primitive earth was found in the locality of that name. When science had discovered the peculiar properties of this magnesian ore to polarize the needle and guide the commerce of the world, Galvani, or Volta, or some one of their peers, should have given it a new name, expressive of its most obvious property; a scientific name in place of its historical name.

There is a deal of sense in calling things by appropriate names—names that imply the discovery of some elementary features which identify this substance and that, and distinguish one from another, as the prominent features of the human face distinguish one man from another. When we remember that the facts of science can live only in words, that ideas can be transmitted from generation to generation only through the media of words; we can see the importance of giving to scientific terms the fullest and completest expression of meaning. In this way only can those coming after us learn of us and comprehend, at a glance, the nature of the facts which we pretend to have discovered.

In the choice of words for the expression of scientific facts, certain obvious rules should be observed. The cognate sciences, anatomy, physiology, pathology, medicine and surgery, which had developed a good degree of maturity before Dentistry was named as a science, are not wanting in a vocabulary of meaning terms from which Dentistry may with much advantage borrow. Dental science, so nearly allied to those which are accounted the noblest and the worthiest that have ever engaged the thought of man, may well feel proud enough of its relations to desire to perpetuate the family names. Here is one chief source of Dental names. Wherein these cognate sciences fail to furnish our Dental vocabulary, by reason of discoveries which reach beyond their scope, we are at liberty to borrow from other sciences where there exist facts so strikingly analogous to the facts of Dental science, as to make it eminently appropriate to use the same form of words in expressing both. This is our second legitimate source of the derivation of Dental terms.

A third means of supplying our Dental vocabulary is by compounding words of definite meaning in general science and art, to form a more complete idea of something in our particular science. A fourth, and the last which I mention, is the coining of new words. General literature, with its varied aims and almost endless variety of methods of arriving at its results, giving unrestrained liberties of trope and figure, may legitimately use new coined words, leaving the meaning to be inferred from the general drift and the connection of thought. But the temple of science can be built only of hewn stone. Words must be sharply defined, and possess a well understood significance, before they are enshrined as the symbols of our thought, or made the monuments of our cherished ideas. Science will never allow the coining of words. Its aim is to teach. Scientific words must, therefore, have in their elemental structure, at least, a definite meaning.

Among all the sciences, chemistry, though faulty still, furnishes us the most systematically formed vocabulary of meaning terms. Words are there made to sparkle with the prominent facts of the science. Pronounce the word *oxygen*, and one of the most commonly observed facts of chemistry is revealed in the name. *Oxygen* is from a Greek word which signifies the acidifying principle. The tendency of the oxygen of the atmosphere to develop acid in so great a variety of substances in common use, led to the giving to this gas the name it bears. The gases early discovered were colorless. When a peculiar gas, colored green, was discovered, the peculiarity of color gave to it the name *chlorine*, which signifies green. These two words serve to illustrate the principle in nomenclature of giving to substances such names as declare one or more of their most observable qualities or properties.

In Dentistry this principle is measurably carried out. Our professional name *Dentist*, and many derivatives from the same root, *dens*, are legitimate and truthful. So also are the names of the different teeth, based upon their formation and uses. The exception in the case of naming the teeth, in a scientific point of view, is in the name of the last tooth developed, commonly called the wisdom tooth, *dens sapientia.* Better to follow the analogy of nomenclature and call it the third molar.

The word *tartar* is objectionable as a scientific term by reason of its untruthfulness, conveying the idea of an acid formation. The term originally applied to the deposit of tartaric acid on the inner surface of wine casks. To call it *calculus* seems a better naming for the reason that it conveys a truthful idea of its principal constituent, *lime.* *Salivary calculus* renders the name more complete by representing its concretionary formation from and with the fluids of the mouth.

The term *erupt* is a disgusting word, and should have no place in our vocabulary as a representative of a physiological process. It belongs to pathology, and should never be borrowed for our Dental science. For what is its signification? Its original signification is a sudden breaking forth and emission of any thing from confinement, as of lava and flame from a volcano, being peculiarly applicable to fluids and semi-fluids. In its application to medical science we have the name *eruptive fever*, very appropriately so called. *Eruptive humors*, the breaking forth and excretion of humors on the skin, as in measles and small-pox. Why transfer this disgusting thought to the pleasing sight of the development and emergence of the human teeth? These latter terms express our meaning, implying a gradual expansion and growth, and starting into view. The teeth come up in striking analogy to the coming up of grain and other seeds. But who would think of speaking of an *eruption* of a field of corn? Or an *eruptive* melon patch?

The word *fang* is similar to the word erupt in its unpleasant and revolting associations, and is further objectionable in the anatomical and physiological falseness of the idea it conveys.

Some years since I penned a criticism for the *Cosmos*, on the use of this same word. For the sake of making this article more complete, I shall be excused for repeating, in part, what I then wrote: when and by whom this word was so unwarrantably foisted into medical and Dental literature does not appear.

*Fang* is a common word of well defined meaning, borrowed from Natural History, but in utter violation of all rule and license. It has in its signification something frightful and repulsive, as a something pointed to thrust, seize, lacerate, rend, or poison. It is proper to call the tusks of a wild bear or wolf, or the poison teeth of a serpent, *fangs*. It is also proper to call the talons of birds of prey, of cats, and of lions, *fangs*. But who has ever perceived any appropriateness in applying this old Saxon word with its repulsive meaning to that which is more familiarly known as the *root* of a tooth? With much greater propriety might we call the instruments with which it is extracted, *fangs.* The term *root* may or may not be the best term to name that portion of a tooth found within the maxilla. It is certainly not inappropriate in its signification. For like the root of a tree, it is both a functional and a mechanical support. Like the root of a tree, it is covered and excluded from external influences, but holds vital relations with its immediate surroundings. Like the root of a tree or plant, it has absorbents, vessels, tubuli through which pass and repass the nourishing and waste fluids. Like the root of a tree, it supports the body of the tooth in firm position against any power tending to topple over or dislodge it. A child turning from his lesson in Natural History might well shudder at the account of *fangs* in the masticatory organization of one of human kind, and very naturally conceive the operation of extracting these to be like the dangerous effort to snatch out the poison teeth of a serpent, or break the weapons of a wild boar.

*Pulp* is another word in such general use by the profession, that it seems now almost too late to ostracise it, or even to criticise it. But as we are favored with other names of the same organ which indicate its alliance with a grand and wonderful system, I hope that prejudice and precedent will not bind us too strongly in favor of an objectionable term. Its primitive signification is that of something disorganized, soft, compressible, and yielding readily to the touch. Led by this signification, semi-fluid and fatty substances are called *pulp.* The material of which paper is made, when ground up into shreds, and mingled with a watery sizing, is called *pulp.* An apple in a state of decay is called pulp. No one will dispute this literal signification of the term, nor its peculiar appropriateness in the cases above mentioned.

The objection which I urge to its use as a Dental term is that it degrades the nature of the organ which it names, by robbing it of its vital functions and its important relationships. The delicate, sensitive, highly organized nerve tissue, given the pre-eminence of a seat in a beautifully built house of ivory, overlaid with pearl, is almost the only vital organ with which we as Dentists primarily have to do. Its obscure retreat, and its almost microscopic line of connection with any important system of the human organism, was unfavorable to the early investigation of its nature and relations. The Dental organs have asserted no special claim to science, and had taken rank not at all as separate organs, but only as a part of the bony structure protruding through the fleshy covering of the body, a part of the bony framework which, like some of the framework in the ruder styles of Gothic architecture, remained uncovered and exposed to view. Its alliance to the bones of the body being *thus* regarded, it is natural enough that the contents of the tooth-bone should be regarded like the contents of other bones, as *marrow*, *pulp*. But when, on later investigation, it was discovered that the teeth had a claim of superiority to other bones, so distinct in their substance and nature as to be no longer called bone, but *dentine*, it was also discovered that the central cavity of the teeth was occupied by a distinct organization, differing almost infinitely from the dead fatty matter centrally located in other bones. Why then, in the name of science, could not this insignificant, degrading word *pulp* have been dropped? Why not call the brain *pulp?* Why do we not say of this or that distinguished man, that he has a well developed *marrow*, a very intellectual *pulp?* For the plain reason that it sounds ludicrous, that it belittles the conception which we have gained of the nature of the organ, that it vulgarizes the crowning organ of man's physical nature to mere gelatinous fat.

Another false, objectionable word has crept in through the Dental laboratory; has been borrowed from anatomical science to grace our glossary of mechanical terms; has arrived at the pinnacle of unquestionable right and authority by occupying a place in our Dental Dictionary: the word is *articulate*. The use of this word has one and only one correct application in mechanical Dentistry, and that is to the union of the teeth to the base plate. *That* joining may, with some propriety, be called an articulation. But it is almost never thus applied. Nothing can be more improper than to speak of the occlusion of the teeth upon each other as an *articulation.* In both anatomy and botany, where the word is at home, it means a union by joints. The different methods of joining named *diarthrosis*, *symphysis*, &c., are different methods of articulation. But in every case it implies a *union*, either movable or fixed. The error, however, in the use of this word is comparatively of slight importance.

In adding new words, and in making modifications of words, great caution is needed so as not to multiply terms needlessly. We do need the introduction of a few new terms to cut short a circumlocution of words now in use to express simple processes, such as the passing of fluids through a porous body. When in the decay of a tooth the mineral portion has been dissolved, leaving the animal portion, a porous structure through which the fluids of the mouth pass to irritate the nerve, a word ready formed which expresses this is *osmose.* And the passing out from the nerve cavity of fluids or gases through such porous structure is well expressed by the correlative term exosmose.

*Dentogeny* is a faithful word signifying the *formation of the teeth.*

*Dentography* is perhaps a needed word, meaning a *description of the teeth.*

*Dentification* for ossification is a desirable change of terms, preserving fully the analogy of a large class of scientific terms already generally adopted by the profession. Another desirable change is *peridenteum* for *Dental periosteum.*

The form of words *mallet pressure* is a contradiction of terms. The word *impact* expresses the idea fully, meaning *condensation by light quick blows.* Thus an impacted filling is one made by light blows of the mallet. Thus far will suffice in scanning the faults and needs of our Dental literature.

I cannot conclude this report on *Dental nomenclature* without uttering a protest in the name of Dental science and literature, against the useless multiplication of technical terms, against cumbering the science with new-coined and meaningless words, against mystifying primary and simple truths with the phraseology of half fledged thought; against putting into the crucible with the refined gold of our science, all the crudities of other sciences, together with the base ores of addled brains too worthless to be accounted as even the dross of good metal. It is to be feared that the editorial corps of our standard literature have yielded too far in their respect for varied learning, and for enthusiasm and zeal in the pursuit of science to dare to sift the pages of their worthy contributors, sending their chaff to the winds and preserving only the sound wheat for their garners.

Some of our most gifted writers seem to be more gifted in words without meaning than in words with meaning, more gifted in befogging a truth than in shedding light upon it, more gifted in mystifying that which before was plain than in bringing into distinct view that which was before obscure or hidden. In an article of several pages published not long since, in one of our standard Journals, there occurred as many words as there were pages, which were nowhere to be found in Webster, Harris, Hooper or Dunglison! Into what depths of lore must the writer have gone to find those mysterious terms! What a balloon ascension must he have taken to crown his topmost ideas with those out of sight words! The soundest learning is found in words having a well understood meaning. If we would preserve our literature from ridicule, if we would hand it down to posterity as the garner house of what is known to the Dental profession, and what is sought after, we must keep out of it what we too often find, and which we can most fitly characterize as conglomerate thought, befogging phraseology, mysterious terms, hair-splitting theories, commingling of science with all the ancient *ologies* and *isms* that have bewildered mankind from the foundation of the world, and instead give to its pages a clear and lucid presentation of what is desirable for us to know and posterity to learn.

